# Recurrent sepsis and neuroinvasive disease in a neonate culture-positive for a Group B Streptococcus CPS III serotype, *hvgA*+ strain

**DOI:** 10.1099/jmmcr.0.005034

**Published:** 2016-06-25

**Authors:** Sneha Suresh, Gregory Tyrrell, Areej Alhhazmi, Sandra Escoredo, Michael Hawkes

**Affiliations:** University of Alberta, Edmonton, Canada

**Keywords:** GBS, meningitis, recurrence, HvgA protein, neonate

## Abstract

**Introduction:**

Late-onset disease with Group B Streptococcus (GBS LOD) remains a significant problem in neonates. Unlike early-onset disease, rates of GBS LOD have not changed with prenatal testing. Effects of GBS LOD can be severe and thus identifying risk factors for severe GBS LOD, such as hypervirulence genes, may help in managing these infants.

**Case presentation:**

We present a case of a neonate with capsular serotype III GBS sepsis without meningitis that recurred 6 days after a 10-day-treatment period with IV ampicillin. The second episode was characterized by sepsis, neuroinvasion, meningitis and subsequent profound encephalomalacia. The short duration between the two episodes suggested recrudescence rather than reinfection. The GBS isolate was ultimately found to be positive for hypervirulence gene *hvgA+*, which encodes for a protein known to mediate meningeal tropism and neuroinvasion.

**Conclusion:**

*hvgA* positivity may thus potentially serve as an important biomarker for severe and neuroinvasive GBS LOD that can influence treatment decisions.

## Introduction

Since the 1960s, *Streptococcus agalactiae* or Group B Streptococcus (GBS) has remained an important bacterial pathogen in neonates (CDC, 2010). Although intrapartum antibioprophylaxis for GBS-positive women has decreased the incidence of early-onset disease (EOD, onset <7 days of age) from about 1.7 per 1000 live births to ~0.3–0.4 per 1000 live births since these programs were introduced, rates of late-onset disease (LOD, onset ≥7 days of age) have remained relatively stable at approximately 0.3–0.4 per 1000 live births (CDC, 2010). Previous studies have implicated the hypervirulent GBS adhesion protein HvgA encoded by the *hvgA* gene as a critical virulence factor for neurotropic neonatal GBS infections (Tazi *et al.*, 2010). We present a case of LOD recurrent sepsis and neuroinvasive infection by a GBS capsular polysaccharide serotype (CPS) III isolate harboring *hvgA* in a preterm neonate with no obvious source for reinfection.

## Case report

A G7, TA1, SA2, P4 mother was admitted to the antepartum unit for 15 days with preterm premature rupture of membranes and an antepartum bleed. She had a previous history of preterm delivery but no prior history of GBS colonization. Her prenatal serology was Hepatitis B Surface Antigen-, Syphilis EIA- and HIV EIA-negative, Rubella IgG immune (30 IU ml^−1^), and Varicella Zoster IgG-positive. A vaginal swab in the first trimester was positive for *Ureaplasma urealyticum*. Vaginal/anorectal swabs and urine cultures done throughout pregnancy did not identify GBS. She received antepartum antibiotics for preterm premature rupture of membranes according to the Mercer protocol, consisting of 48 h of IV ampicillin (2 g q6h) and erythromycin (250 mg q6h) followed by oral amoxicillin (250 mg q8h) and erythromycin (333 mg q8h) for 5 days (Mercer *et al.*, 1997).

Fifteen days after admission, at 27 + 1 weeks gestation, the mother was noted to have umbilical cord prolapse and was sent for emergency C-section. There was no maternal fever. Placental pathology was consistent with acute chorioamnionitis and umbilical cord vasculitis.

The infant was born with Apgars of 5, 8 and 8 at one, five and ten minutes, respectively. The birth weight was 1050 g (50–90^th^ percentile), head circumference was 25 cm (50–90^th^ percentile), and length was 37 cm (50–90^th^ percentile). The infant required positive pressure ventilation during the resuscitation. Throughout his NICU stay his major issues were respiratory distress syndrome (managed with surfactant, CPAP and high-flow nasal cannula), apnea of prematurity (managed with caffeine) and neonatal jaundice (managed with phototherapy,) and was worked up slowly from TPN to enteral feeds. The infant solely received donor human milk and formula as enteral nutrition.

At birth, the infant had a blood culture and CBCD done and was started on ampicillin 55 mg (50 mg kg^−1^ per dose) q12h and gentamicin 5.5 mg (5 mg kg^−1^ per dose) q48h. The initial CBC had a normal haemoglobin and platelets and white blood cell count of 4.1 × 10^9^l^−1^ and neutrophils of 0.9×10^9^ l^−1^. Initial blood cultures were negative at 48 h and antibiotics were discontinued at that time.

At 21 days of age, the infant became hypotensive with signs of systemic shock. Cultures of blood, urine and cerebro-spinal fluid (CSF) samples were performed. Cloxacillin 60 mg (50 mg kg^−1^ per dose) q8h and gentamicin 6 mg (5 mg kg^−1^ per dose) q24h were empirically started for neonatal sepsis. Blood culture revealed Gram-positive cocci in chains at 8 h incubation and cloxacillin was substituted for vancomycin 12 mg (10 mg kg^−1^ per dose) q12h for empiric coverage. Definitive cultures ultimately revealed the isolate as GBS CPS III and the following day the infant was switched from vancomycin to ampicillin 120 mg (100 mg kg per dose) q8h. Urine culture was also positive for 10^2^ CFU ml^−1^ GBS. CSF culture, done after one dose of cloxacillin, one dose of gentamicin and one dose of vancomycin was negative for bacterial growth after 5 days incubation. Cell count and chemistry revealed a WBC count of 3 × 10^6^ l^−1^, RBC count of 5 × 10^6^ l^−1^, glucose at 3.5 mmol l^−1^ (no serum done at time) and protein at 1.14 g l^−1^. The WBC count was too low to perform a differential. Blood cultures were negative the following day and the episode was treated as a GBS sepsis with intravenous ampicillin 120 mg (100 mg kg^−1^ per dose) q8h and gentamicin 6 mg (5 mg kg^−1^ per dose) q24h for 10 days from the first negative blood culture. A cranial ultrasound done after nine days of therapy showed no ventricular dilatation, bleed or intraparenchymal abnormality, and was reported as normal.

**Fig. 1.**
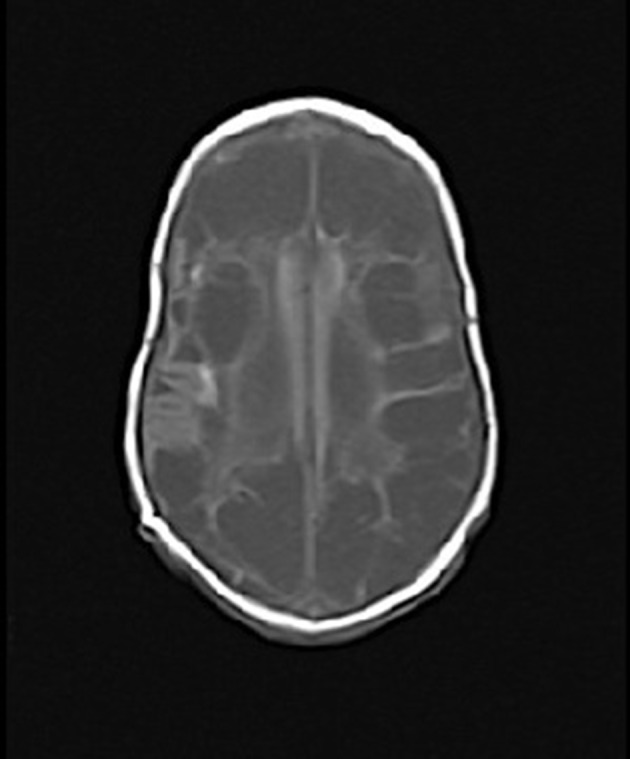
MRI of brain of infant 3 weeks post-second episode of GBS sepsis.

Six days after discontinuation of antibiotic therapy, the infant became apneic, grey, pale and with bounding pulses and poor perfusion. The infant required fluid resuscitation, intubation, ventilation and inotropic support. Repeat blood and urine cultures were obtained, and meropenem 64 mg (40 mg kg^−1^ per dose) q8h and gentamicin 8 mg (5 mg kg^−1^ per dose) q24h empiric therapy was started. The second blood culture revealed Gram-positive cocci in chains at 4 h, at which vancomycin 16 mg (10 mg kg^−1^ per dose) q8h was again empirically started. The blood culture isolate again identified as GBS CPS III. Lumbar puncture was performed the following day after clinical stability. Urine culture at this time was negative as well as CSF cultures (obtained after antibiotics were administered). CSF cell count analysis revealed >5000 RBCs, 145 WBC with a differential of 56 % neutrophils, 36 % monocytes/macrophages, 2 % eosinophils and 2 % lymphocytes. Protein and glucose were not measured given blood contamination of CSF. A search for a nidus of infection was undertaken, including echocardiogram, abdominal ultrasound and skeletal survey, all of which were unremarkable. Cranial ultrasound done two days after therapy was started showed no cystic change, ventriculomegaly, subependymoma or interventricular haemorrhage, and was reported as normal. Two days after the septic deterioration, inotropes were weaned off, definitive cultures and susceptibility were back, and the infant was stepped down to ampicillin 160 mg (100 mg kg^−1^ per dose) q8h with a planned 3-week course for GBS meningitis. An MRI brain scan done at the end of three weeks of therapy revealed severe symmetrical bilateral supratentorial cystic encephalomalacia involving the frontal, temporoparietal and occipital lobes (Fig. 1). Areas of restricted diffusion were concerning for ongoing ischemic or inflammatory changes of the little remaining normal brain parenchyma. There was relative sparing of the brainstem and posterior fossa. Repeat CSF analysis revealed macroscopic blood (not quantified) and WBC count of 444 × 10^6^ l^−1^ with a differential of 61 % neutrophils, 25 % lymphocytes, 12 % monocytes/macrophages and 2 % eosinophils. Given the imaging and CSF findings, the decision was made to treat the infant for complicated meningitis for three more weeks, with a total 6-week course of ampicillin 230 mg (100 mg kg^−1^ per dose) q6h. Remarkably, the infant did not have any seizure activity, and continued to progress from nasogastric to bottle-feeds. He was noted to have an abnormal left auditory brainstem reflex. Close developmental follow-up was planned after NICU discharge at 101 days of life, with a corrected gestational age of 41 + 4 weeks.

## Investigations

Both GBS isolates (at 21 days and 37 days) were found to be CPS III isolates, with similar antimicrobiograms (resistant to erythromycin, inducible resistance to clindamycin, and ampicillin-, penicillin-, vancomycin- and cefotaxime-susceptible). Serotyping was performed as previously described (Haug, 1972; Lancefield, *et al.*, 1975). As this was a neurological case of invasive GBS LOD and the HvgA protein has been previously demonstrated to be involved in meningeal tropism in neonates, PCR targeting the *hvgA* gene was done as previously described (Tazi *et al.*, 2010). Both isolates were *hvgA*-positive.

## Discussion

This case demonstrates the devastating potential of neonatal GBS disease. Though this particular patient had risk factors for EOD (chorioamnionitis, prematurity, prolonged rupture of membranes) cultures done at birth did not reveal evidence of early GBS infection, and rather the infant developed LOD at 21 days of age. Recrudescence, rather than re-infection, was suspected on the basis of the short time between cessation of antibiotic therapy and repeat infection, together with identical genotypic and phenotypic features of the GBS isolates. The site of subclinical persistence under this hypothesis is unclear, but elevated CSF protein (without pleocytosis) during the first septic episode may point to under-treated meningitis as a potential source.

Both host susceptibility and virulence of the pathogen contributed to the severity of this illness in the patient. With regards to organism virulence factors, the isolate was CPS III GBS strain. CPS III is a common serotype for LOD and is the most prevalent serotype seen in Alberta, Canada for LOD (>60 % of LOD) (Alhhazmi *et al.*, 2014). In addition, this strain was positive for *hvgA*. HvgA is a specific surface anchored protein first characterized in a hypervirulent ST-17 clone of GBS (Tazi *et al.*, 2010). HvgA promotes adherence to intestinal epithelial cells, choroid plexus epithelial cells and microvascular endothelial cells, and is thought to be a critical virulence factor for neurotropic strains of GBS disease (Tazi *et al.*, 2010).

With regards to risk factors for LOD, a prospective cohort study of LOD GBS infants from 2003–2010 showed an association with maternal mastitis and concurrent rectovaginal GBS carriage (Berardi *et al.*, 2013). Intrapartum antibiotics modulated disease severity (Berardi *et al.*, 2013). Breast milk has also been implicated in the pathogenesis of LOD, and especially in recurrent infection with one review of 48 cases of LOD identifying clonal isolates in both the neonate and mother’s milk with a recurrence rate of 35 % (Filleron *et al.*, 2014).

Host factors have also been implicated in LOD. Prematurity is a risk factor for both EOD and LOD, as the majority of protective maternal antibody is not thought not to pass until the third trimester of pregnancy (Lin *et al.*, 2003). HIV exposure has also been implicated as a risk factor for LOD, as well as the primary immunodeficiency IRAK 4 mutation (Krause *et al.*, 2009; Epalza *et al.*, 2010).

Other than prematurity, there were no other obvious risk factors for LOD or recurrent disease noted in our patient (infant not breast fed, mother received intrapartum antibiotics). Though prematurity remains a significant risk factor in this case, the absence of other characterized routes of transmission in the neonate, and the fact that this neonate had both recurrence and destructive brain pathology from this infection speaks to the hypervirulence of this particular strain of GBS.

It is possible that in our case the initial episode of GBS infection was mischaracterized as bacteremia without meningitis on the basis of absent pleocytosis. Previous studies have suggested that the use of pleocytosis (CSF >21 cells mm^−3^) for diagnosis of neonatal bacterial meningitis has a sensitivity of 79 % and specificity of 81 % (Garges *et al.*, 2006). Though culture is considered the gold standard, CSF for the diagnosis of bacterial meningitis in neonates is often obtained after antibiotic administration, and pleocytosis, in the absence of other clinical or imaging findings, is often the only available tool to diagnose a CNS infection. The possibility that a CNS infection might have been occurring in our infant despite a low CSF WBC count speaks to the need of studying other pathogen-related factors that may be able to predict CNS involvement.

Identifying GBS-infected infants at risk of recurrent or neuroinvasive disease may be challenging, as in our case with no CSF pleocytosis and normal brain ultrasound at the first episode of sepsis. With local rates of GBS LOD recently holding at 0.39 per 1000 live births, continued study of this disease is integral to infant health. *HvgA* PCR is currently only available in specialized research laboratories and has not been been implemented for routine clinical use in Canada that we are aware of. Thus our center has not made any recommendations regarding its clinical utiity. HvgA deserves further study as a potential biomarker of hypervirulence that could guide clinical management, by escalating vigilance, ancillary testing, treatment duration, and/or neurodevelopmental follow-up.
